# Educational Interventions to Promote Cervical Cancer Screening among Rural Populations: A Systematic Review

**DOI:** 10.3390/ijerph19116874

**Published:** 2022-06-04

**Authors:** Mengyue Zhang, Janet W. H. Sit, Dorothy Ngo Sheung Chan, Oluwadamilare Akingbade, Carmen W. H. Chan

**Affiliations:** The Nethersole School of Nursing, Faculty of Medicine, The Chinese University of Hong Kong, Hong Kong 999077, China; kassiezhang@link.cuhk.edu.hk (M.Z.); janet.sit@cuhk.edu.hk (J.W.H.S.); dorothycns@cuhk.edu.hk (D.N.S.C.); oakingbade@link.cuhk.edu.hk (O.A.)

**Keywords:** cancer screening, health education, uterine cervical neoplasm, systematic review

## Abstract

The urban–rural gap in cervical cancer screening uptake is a significant public health consideration. Educational interventions are commonly adopted to promote cervical cancer screening among females in rural areas; however, the characteristics and effectiveness of these educational interventions remain unclear. In this review, we aimed to identify the characteristics of educational interventions used in rural populations and to evaluate the effects of these interventions on cervical cancer screening-related outcomes. Seven English databases were searched in January 2022. Randomized controlled trials (RCTs) and quasi-experimental studies were included. The Joanna Briggs Institute (JBI) Critical Appraisal Checklist for Randomized Controlled Trials and the JBI Critical Appraisal Checklist for Quasi-Experimental Studies were used for quality appraisal. RevMan 5.4 software was used for the meta-analysis. A narrative synthesis was conducted in instances where a meta-analysis was inappropriate. Three RCTs and seven quasi-experimental studies conducted in six countries were included. A social cognitive theory-based framework, the community setting, group sessions, healthcare professional-led approaches, and culture-tailored materials were implemented in the educational interventions for cervical cancer screening. The educational content mainly included basic information on cervical cancer screening, psychological issues, barriers and strategies to overcome them, and locally available resources. Educational interventions increased the knowledge and uptake of cervical cancer screening in the rural population. However, the studies only evaluated the short-term effects of these educational interventions, with the cervical screening behavior only being assessed in one instance for each participant. Educational interventions promote cervical cancer screening among females in rural areas. Theory-driven, community-involved, group-based, and healthcare professional-led approaches should be prioritized in the application of educational interventions in rural populations. Both the short- and long-term, influences of educational interventions on the cervical cancer screening behavior of females in rural areas need to be recognized.

## 1. Introduction

Cervical cancer is a significant global health issue. In 2020, cervical cancer ranked fourth among the top five most frequently diagnosed cancers in females [1]. Previous studies have reported urban–rural disparities in the incidence and mortality of cervical cancer, and the disease burden of cervical cancer in rural areas is significantly higher than in urban areas. From 2009 to 2013 in the US, the incidence of cervical cancer in rural areas was about 1.15 times that of urban areas, while the mortality was about 1.13 times. In China during 2010, the incidence of cervical cancer in rural areas was about 1.44 times that of urban areas, while the mortality was about 2.47 times [2,3]. As well as socioeconomic differences, other urban–rural health disparities have been identified, including insufficient healthcare resources and a poor health status in rural areas [4,5,6,7]. Therefore, reducing the disease burden in rural populations is important. To reduce the gap in urban–rural cervical cancer control, it is essential to prioritize cervical cancer prevention in rural areas.

As an effective secondary prevention strategy, screening is important for the early detection of cervical cancer. Females who are at a high risk of cervical cancer can be identified using various screening methods, and timely follow-up treatment can be arranged [8,9]. Hence, it is crucial to promote cervical cancer screening among rural populations for cervical cancer prevention. However, studies from different countries have identified lower cervical cancer screening rates among rural populations than their urban counterparts [10,11,12,13,14,15]. To promote cervical cancer screening in rural populations, effective interventions that are suitable for use in rural areas and that apply to the needs and challenges of healthcare practices in rural areas should be developed and implemented. 

Previous studies have reported that an inadequate awareness of cervical cancer and screening is one barrier to the uptake of cervical cancer screening in rural areas [16,17,18,19,20]. Educational interventions refer to health education activities that aim to positively improve people’s health-related knowledge and awareness and thus change the relevant behavior [8]. Evidence shows that educational interventions can promote cervical cancer screening. A systematic review of studies that included females in both urban and rural areas evaluated the effectiveness of interventions on increasing the uptake of cervical cancer screening. These interventions had positive effects on increasing screening uptake [21]. Another systematic review focused on health educational interventions for females in both urban and rural areas, and revealed that educational interventions effectively increased the uptake of cervical cancer screening [22]. One scoping review focused on summarizing interventions on increasing cervical cancer screening uptake reported that educational interventions are commonly implemented in rural areas [23]. 

However, all of the above reviews only identified educational interventions that effectively increased cervical cancer screening, without exploring which interventions were efficacious according to the format, delivery mode, and educational content. One systematic review examining educational interventions for cervical cancer screening investigated the effective education type according to the education format and materials [24]. This review included females in both urban and rural areas [24]. However, considering the low level of socioeconomic development [6,7], the inadequacy of healthcare resources in rural areas [25], and the low health literacy among rural populations [26], the characteristics of educational interventions for use in rural populations might be distinctive and unique, and the effects of these interventions might also be diverse. Therefore, to better implement effective educational interventions to promote cervical cancer screening among females in rural areas, it is necessary to identify the characteristics of educational interventions for use in rural populations and to evaluate the effectiveness of these interventions. This would expand the current understanding of intervention strategies in terms of their essential components, delivery formats, acceptability, sustainability, and effectiveness specific to the cervical cancer screening-related outcomes of females in rural areas. 

Until now, to the best of our knowledge, no systematic reviews or meta-analyses on this topic have explicitly focused on rural populations. Previous studies have determined that educational interventions effectively improve cervical cancer screening uptake among females in urban and rural areas [22,23,27] and discussed effective educational intervention models for use in the general population [24]. Nevertheless, the characteristics and effectiveness of educational interventions on cervical cancer screening in rural populations remain uncertain. Therefore, this systematic review aimed to identify the characteristics of educational interventions for use in rural populations and to evaluate the effectiveness of these interventions in promoting cervical cancer screening from an integrative perspective. 

## 2. Methods

### 2.1. Data Search and Sources

We conducted a systematic literature search in January 2022. The Population, Intervention, Comparison, and Outcome framework was used to generate the search strategy. Seven databases (MEDLINE, EMBASE, PubMed, Web of Science, CINAHL Complete, Global Health, and Cochrane Central Register of Controlled Trials), as well as the ClinicalTrials register, World Health Organization website, and reference lists of identified articles, were searched. The search items included ‘cervical cancer’, ‘cancer screening’, ‘education’, and ‘rural’, which were searched in all possible combinations and with Medical Subject Headings.

### 2.2. Study Inclusion and Exclusion Criteria

Interventional studies, including randomized controlled trials (RCTs) and quasi-experimental studies published in English with no publication time limitation, were included. Reviews, conference abstracts, protocols, and ongoing trials (without reported results) were excluded. In terms of the participants, studies that recruited females aged ≥18 years in rural areas who were eligible for cervical screening were included. This review focused on cervical cancer screening among the general population in rural areas. Therefore, studies that targeted participants with any diagnosis of severe physical or mental illness were excluded. The included interventions were cervical cancer screening-related educational interventions directly provided to females in rural areas. The primary outcome was cervical cancer screening uptake among females in rural areas, regardless of the screening method used, such as Papanicolaou (Pap) smear, visual inspection with acetic acid (VIA), and human papillomavirus (HPV) testing. The secondary outcomes included the knowledge, attitude, and intention to undergo cervical cancer screening. Studies that reported at least one of the above-mentioned outcomes were included. 

### 2.3. Study Selection

The Preferred Reporting Items for Systematic Reviews and Meta-analyses (PRISMA) guidelines were used to develop this review [28]. EndNote X9 (Clarivate Analytics, PA, USA) was used to manage the identified studies, and two reviewers screened the studies independently using the PRISMA flow. Any disagreements between reviewers were resolved by consensus.

### 2.4. Quality Appraisal and Data Extraction

Joanna Briggs Institute (JBI) Critical Appraisal Tools were adopted to assess the methodological quality of the included studies. The JBI Critical Appraisal Checklist for Randomized Controlled Trials [29] and the JBI Critical Appraisal Checklist for Quasi-Experimental Studies [30] were used, as appropriate. The checklist for RCTs contains 13 items that are intended to evaluate the general quality of studies according to randomization, allocation, blinding, follow-up, and outcome measurement and analysis. The checklist for quasi-experimental studies includes nine items that are intended to evaluate the quality of studies according to the causality of variables, baseline, control, and outcome measurement and analysis. Each item is assessed as ‘yes’, ‘no’, ‘unclear’, or ‘not applicable’. If ‘yes’ is chosen, 1 point is scored. Studies scoring ≥ 6 points were regarded as good quality and were included in the review [31]. Two reviewers completed the quality assessment independently, and any disagreements were resolved by consensus. The data were extracted by use of a self-developed form.

### 2.5. Data Synthesis

RevMan 5.4 software (The Cochrane Collaboration, London, UK) was used for the meta-analysis. The relative risks (RRs) and 95% confidence intervals (CIs) were used to summarize the effect sizes of the cervical cancer screening uptake rate. Cochran’s Q test was used to calculate heterogeneity, and the random-effects model was used when the heterogeneity was statistically significant (I^2^ > 50%). Otherwise, the fixed-effects model was used. A narrative synthesis was conducted for outcomes that were not suitable for pooling into the meta-analysis. 

## 3. Results

**Figure 1:**
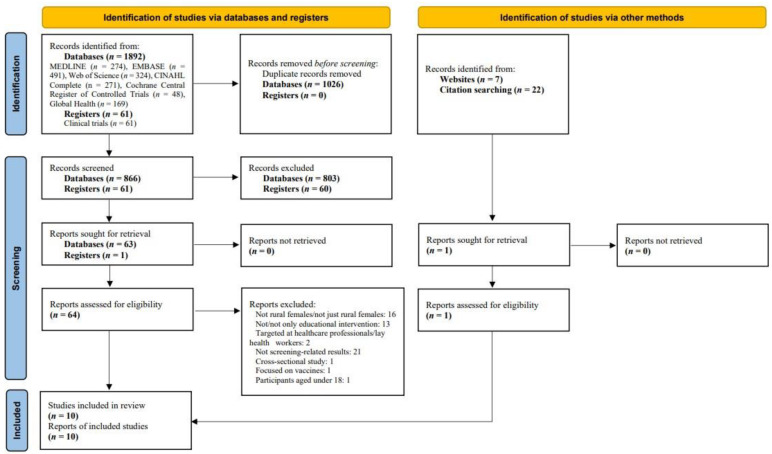
PRISMA flow diagram.

A total of 1953 records were identified from databases and registers, and 29 additional records were identified through the websites and reference lists of the identified articles (Figure 1). After removing duplicates and abstract screening, 65 reports remained for full-text screening. Of these, 10 studies published between 2011 and 2021 were eligible for inclusion in this review [32,33,34,35,36,37,38,39,40,41]. Four studies [32,34,36,38] were conducted in developed country (the US), while six studies [35,37,38,39,40,41] were conducted in developing countries (Cameroon, Nigeria, Iran, Malawi, and India). Among the 10 studies, three [32,33,34] were RCTs and seven [35,36,37,38,39,40,41] were quasi-experimental studies.

### 3.1. Quality Appraisal

Three RCTs scored 7 points [32], 8 points [33], and 10 points [34], respectively. Reporting randomization and blinding were vital when assessing the methodological quality of the RCTs (Table 1). However, only one RCT explained the randomization method [33], and one study mentioned the blinding method [34]. Of the seven quasi-experimental studies (Table 2), two single-group pre- and post-test studies [40,41] scored 7 points. One study that used a control group [35] scored 7 points; however, it did not mention the intervention that was used in the control group. Moreover, it did not explain the missed follow-up. One study [36] with a control group scored 8 points, which was also because the control intervention was unclear. The other three controlled studies, which were of high quality, scored 9 points [37,38,39].

### 3.2. Characteristics of the Study Population

A total of 2901 females in rural areas were included in the 10 studies (Table 3). The age of the participants varied among studies, with the majority ranging from 20–60 years of age. One study [32] only targeted females aged >50 years. The educational level of the participants ranged from primary school or lower to college or university, with most participants being less educated [32,33,34,35,37,39,40,41]. Only one study targeting immigrant females reported that most rural female participants (73.9%) had college/university education [36]. Cervical cancer screening methods used in rural areas included Pap smear, VIA, and HPV testing.

### 3.3. Characteristics of Educational Interventions for Use among Females in Rural Areas

#### 3.3.1. Theoretical Framework

Six studies developed educational interventions based on theoretical frameworks. Two studies [36,41] were based on the theory of planned behavior. Three studies [32,34,38] adopted the social cognitive theory, with one study [38] combined two theories, social cognitive theory and popular education. One study [37] adopted the Health Belief Model (Table 4).

#### 3.3.2. Intervention Delivery Mode

Group sessions were widely adopted among the included studies. Seven studies [32,33,35,36,37,38,39] reported interventions based on face-to-face group educational sessions. One study [34] adopted individualized interventions with either video or home visits. One study [40] used a digital device-based intervention. With this approach, a tablet was used to deliver interactive education.

#### 3.3.3. Intervention Deliverer

Healthcare professionals (research investigators with a health profession background) were the most common interveners among the studies. In addition, three studies [32,34,38] used a ‘Promotora’, which is a lay health worker, to deliver the intervention.

#### 3.3.4. Settings

The community was the most common intervention setting among the included studies, with six studies [32,34,36,38,39,41] reporting implementation in community-based settings, including participants’ homes, churches, and public places. Two studies [33,37] conducted the interventions at healthcare centers. The other studies [35,40] did not report their intervention settings. 

#### 3.3.5. Educational Content

The educational content in the included studies varied but could be divided into four categories: relevant basic knowledge, psychological issues, barriers and strategies to overcome them, and locally available resources. All studies provided basic cervical cancer screening knowledge, including content on the anatomy and pathology of cervical cancer and screening methods. Meanwhile, three studies [32,33,37] explained psychological issues relevant to cervical cancer screening in their interventions, three studies [34,37,38] mentioned screening-related barriers and methods to overcome them, and seven studies [32,34,36,37,38,40,41] specifically introduced locally available resources for screening (Table 5). 

#### 3.3.6. Educational Materials

Audio-visual materials were widely used among studies, with eight studies [33,34,35,37,38,39,40,41] reporting the use of videos, films, or movies to deliver health education (Table 6). Meanwhile, reading materials, including brochures, pamphlets, booklets, and leaflets, were commonly adopted as supporting materials [34,35,37,38,39]. Four [33,34,35,40] studies emphasized the use of culturally tailored educational materials. One study used the local language to develop a movie describing the local culture and tradition [35]. One study designed a culturally sensitive short video on improving positive attitudes towards cervical cancer screening [33]. One study reported the development of a culturally appropriate video in their local language [34]. One study [40] designed the educational content in the local language and in a culturally appropriate manner. 

### 3.4. Effectiveness of Educational Intervention on Cervical Cancer Screening

#### 3.4.1. Primary Outcome: Screening Uptake

Six studies evaluated the post-intervention difference in the cervical cancer screening uptake rate between groups, including two RCTs [32,34] and four quasi-experimental studies [35,36,37,38]. All of these studies only evaluated one-time post-intervention screening uptake among females in rural areas. Five studies [32,34,36,37,38] evaluated the uptake rate of Pap smear, and one study [35] assessed the uptake rate of VIA (Table 7). 

**Figure 2:**
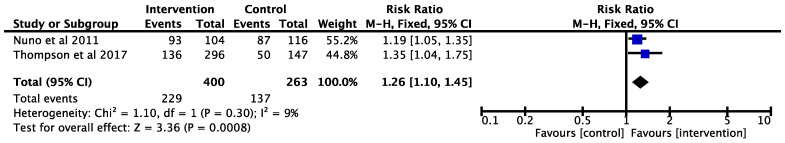
Effects of educational interventions on cervical cancer screening uptake (RCTs).

**Figure 3:**
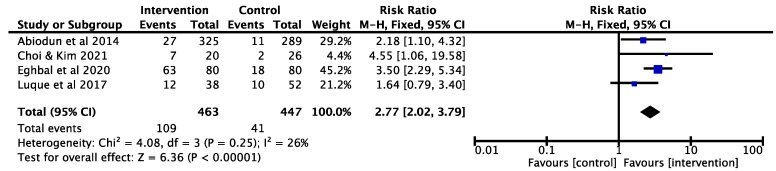
Effects of educational interventions on cervical cancer screening uptake (Quasi-experimental studies).

The meta-analysis was conducted for RCTs and quasi-experimental studies, respectively. The results of the meta-analysis of two RCTs [32,34] demonstrated that educational interventions increased cervical cancer screening uptake (RR = 1.26; 95% CI 1.10–1.45; *p* = 0.0008; I^2^ = 9%; Figure 2). For quasi-experimental studies [35,36,37,38], the meta-analysis results also showed that educational interventions were effective in increasing the cervical cancer screening uptake (RR = 2.77; 95% CI 2.02–3.79; *p* < 0.00001; I^2^ = 26%; Figure 3). 

#### 3.4.2. Secondary Outcomes: Knowledge, Attitude, and Intention

A total of eight studies [33,34,35,37,38,39,40,41] evaluated participants’ knowledge with self-developed questionnaires. The items and measurements included in the questionnaires differed between studies. Because of heterogeneity, the meta-analysis was not appropriate. All studies determined that the cervical cancer screening-related knowledge of females in rural areas increased after receiving the relevant education. One RCT [34] and one quasi-experimental study [39] compared two types of health education, and both reported that regardless of the education type, screening-related knowledge led to different degrees of improvement among females in rural areas (Table 7). 

In terms of screening-related attitude, the outcome measurements varied among studies; hence, a meta-analysis was not appropriate. One RCT [34] and two quasi-experimental studies [37,38] evaluated participants’ self-efficacy in attending cervical cancer screening. One study [37] reported that screening-related self-efficacy improved after participation in the educational intervention. In contrast, two studies [34,38] found no statistically significant post-intervention difference in self-efficacy between the intervention and control groups. Some studies also evaluated screening-related acceptability, willingness, awareness, perception, and intention. However, due to the limited number of studies and the inconsistent results, the effectiveness of educational interventions on cervical cancer screening-related intention and attitude remains unclear (Table 7).

#### 3.4.3. Outcomes by Educational Intervention

##### Group Education and Individual Education

Seven studies [32,33,35,36,37,38,39] reported group-based educational sessions, while one study [34] reported individualized education. Among the studies that used group-based education, five studies [32,35,36,37,38] evaluated cervical cancer screening uptake as the outcome, and four studies reported that screening uptake increased after group education. In contrast, one study [38] reported that the post-intervention screening uptake rate was not significantly different between the intervention and control groups. All group-based interventions positively improved the screening-related knowledge of females in rural areas. Meanwhile, one study [35] found that group education helped to increase the awareness and perception of cervical cancer screening. One study [37] showed that group-based education enhanced screening-related self-efficacy. The study that used individualized education was a three-armed RCT [34], with two types of individualized education. One type was in the format of self-directed learning, and the other was in the format of a home visit. This study reported that the home visit increased cervical cancer screening uptake, while self-directed learning did not. These two types of individualized education also improved screening-related knowledge, but no significant change was observed in terms of screening-related self-efficacy. 

##### Healthcare Professional-Led Education and Lay Health Worker-Led Education

All healthcare professional-led educational interventions [35,36,37,39,40,41] improved cervical cancer screening uptake and knowledge. Screening-related self-efficacy and awareness were also enhanced. Of the three studies [32,34,38] that adopted lay health worker-led education, one study [38] reported no change in the cervical cancer screening uptake rate. Two studies [34,38] evaluated screening-related self-efficacy, but these studies found no significant post-intervention changes among female participants in rural areas.

## 4. Discussion

This systematic review analyzed the characteristics of educational interventions for use in rural populations and evaluated the effectiveness of these interventions in improving cervical cancer screening-related outcomes. The study results provide evidence and insight to support the development of cervical cancer screening-related educational interventions for use in rural populations.

In terms of the methodological quality of the included studies, the general quality was good. However, the most notable issue among the included RCTs was unclear reporting of randomization and blinding methods [32,33,34]. In terms of the quasi-experimental studies with control groups [35,36], unclear reporting of the comparison was noteworthy. Given these limitations, high-quality studies with clear reports of the research methods are needed in the future. 

We identified that most females included in this review had a limited level of education, with many only being educated to primary school level or below. This deficient educational background might have influenced the learning ability and health literacy of these participants. Previous studies have reported that poor general literacy might cause poor health literacy [42,43] and that health literacy among rural populations is low [26]. Therefore, in terms of the development of educational interventions for use in rural populations, evaluating the educational background of the participants and developing appropriate interventions according to their general literacy and health literacy are essential.

Many of the included studies adopted a theoretical framework to develop the educational interventions. In this review, we found that the social cognitive theory was commonly adopted, with three [32,34,38] of six theory-based studies adopting this as the theoretical framework for intervention. The social cognitive theory explicitly explains the complex process of changes in human behavior [44], which helps to provide a stable foundation for developing interventions that can change human behavior [45]. The theory of planned behavior and the Health Belief Model were also used as theoretical frameworks. Our findings are consistent with a previous study, which demonstrated that these three frameworks are frequently used in cervical cancer screening education programs [24]. A previous study reported that adopting a theoretical foundation is essential for developing interventions to promote cervical cancer screening [23]. In addition, one study reported that compared with non-theory-based interventions, the key components and their interrelationships were clearer in theory-based educational interventions [46]. In the current review, the included studies that used theory-based educational interventions did not report the application of theory components in intervention development. However, three studies [34,37,38] incorporated an evaluation of some screening outcomes identified from intervention structures as self-efficacy. Among these, one study found that theory-based intervention was effective for improving these outcomes [37]. Hence, in future practice, to implement educational interventions to promote cervical cancer screening in rural populations, theories relevant to changes in behavior could be adopted as frameworks. Moreover, theoretical constructs could be applied to generate intervention components and outcome measurements.

Face-to-face group sessions were the most widely used intervention delivery mode among the included studies. This approach was effective in promoting screening uptake and knowledge among females in rural areas [32,35,36,39]. A systematic review of cancer screening interventions among rural populations also recommended adopting group educational interventions because of their effectiveness in increasing cervical cancer screening uptake [27]. During group sessions, participants can obtain knowledge from the person delivering the session and interact with other group members. These interactions and the influence of group members might also influence the attitude and behavior of females in rural areas towards cervical cancer screening [47]. In this review, one study [40] adopted digital device-based health education, where interactive educational information was delivered via an electronic tablet. This study showed that digital device-based health education could help to improve the screening-related knowledge of females in rural areas. With the development of health information and communication technology, health education and promotion activities are becoming more widely used, and people can access abundant health information using digital devices [48]. Considering that available healthcare resources are limited in rural areas [25], utilizing digital devices to deliver health education can be beneficial [48]. However, only one study included in this review adopted digital device-based health education; thus, the effectiveness of this educational mode on promoting cervical cancer screening among rural populations requires further investigation in the future. 

In addition to healthcare professionals, ‘promotores’ were also commonly adopted to deliver education. Promotores are community lay healthcare workers who are not professional healthcare workers but who have undergone professional training to deliver related healthcare services, such as educational talks [49]. Lay health workers play a crucial role in rural primary health care as they contribute to undertaking a wide range of health-related interventions [50]. Under the context of health workforce crises, involving lay health workers in health practices has increased [50]. One noteworthy point is that professional training is necessary to prepare competent lay health workers [49]. In this review, we included studies implemented both in developed and developing countries, however, all promotora-led educational interventions in this review were conducted in the US [32,34,38]. Compared with developed countries like the US, lay health worker training might be difficult in rural areas with limited resources. Introducing lay health workers might be important to reduce the workload of healthcare workers and to improve the knowledge and healthcare needs of females in rural areas [51]. However, the cost of training and the resources needed to train competent lay health workers in rural areas also need to be considered [52]. Therefore, in future studies, local healthcare resources and training for lay health workers must be considered when conducting lay health worker-led educational interventions in rural areas. 

Most of the included studies were conducted in community-based settings. A systematic review found that delivery of community-level intervention was vital for cervical cancer screening-related education [22]. According to the World Health Organization [8], community outreach and community mobilization are essential to conduct cervical cancer screening health education. Therefore, considering the rich and varied available community resources and convenience to participants, community-based health education could be provided to rural females in the future [8].

The educational content varied between the included studies, but it was mainly concentrated in four areas: cervical cancer screening-related basic knowledge, psychological issues, barriers, and methods to overcome them, and locally available resources. Most included studies [32,34,36,37,38,40,41] specifically provided local screening-related resources to rural females. Previous studies demonstrated that healthcare resource distribution in rural areas was a significant issue [53], while geographic elements, transportation, and insufficient specialized health organizations were barriers to the access of healthcare services in rural populations [54]. Therefore, providing locally available resources for cervical cancer screening to females in rural areas would be convenient, enabling them to directly understand screening-related services. This could increase the utilization of healthcare resources in rural populations to promote cervical cancer screening. Audio–visual materials and reading materials were widely used as educational materials, and video was a primary educational material among the included studies. Three studies [33,34,35] emphasized the use of culturally tailored videos delivered in local languages, as well as the development of culturally sensitive content. Previous studies [10,20,55] have reported that culture is one significant barrier to participation in cervical cancer screening. Therefore, when preparing supporting educational materials for future studies, these cultural barriers should be considered. Developing culture-tailored materials to promote positive attitudes towards and acceptance of cervical cancer screening will also be important [33]. 

In terms of the effectiveness of educational interventions to promote cervical cancer screening, all of the included studies [32,34,35,36,37,38] only evaluated the post-intervention cervical cancer screening behavior on one occasion without assessing the effectiveness of intervention on promoting regular screening behavior. The meta-analysis results revealed that educational interventions increase the cervical screening uptake of females in rural areas. Cervical cancer screening should be repeated regularly [8]. Thus, evaluating the effectiveness of educational interventions on long-term outcomes, including regular screening habits, needs to be considered. Our findings are in agreement with a previous study [23], which reported that evaluating the effects of interventions on cervical cancer screening uptake should include short-term and long-term outcome measurements.

Some studies chose to adopt ‘intention/willingness’ rather than screening uptake to evaluate the effectiveness of the interventions in promoting cervical cancer screening. The intention was defined as the decision and motivation to formulate health-related behavior. However, many individuals failed to execute their intention [56]. Therefore, an ‘intention–behavior gap’ emerged [56]. Due to this gap, the assessment of intention was regarded as weak in terms of its ability to predict a change in behavior [57]. Therefore, using intention as an indicator of the effectiveness of educational interventions in promoting cervical cancer screening might be deficient. Choosing appropriate outcome measurements is essential.

To assess the influence of educational interventions on the screening-related knowledge of females in rural areas, all of the included studies used self-developed questionnaires. However, most of the included studies did not report the reliability and validity of these self-developed questionnaires. The reliability and validity of self-developed instruments need to be reported clearly and should be examined in future studies. Due to measurement heterogeneity, the influence of these interventions on the knowledge and attitude towards cervical cancer screening could not be evaluated quantitatively. Based on the results among studies [33,34,35,37,38,39,40,41], it may be concluded that educational interventions can improve the knowledge of females in rural areas. However, the effects of these interventions on the attitude towards cervical cancer screening need to be examined further.

We also analyzed screening-related outcomes according to the intervention components. We found that the group-based and healthcare professional-led approaches were popular among studies in this review. Seven studies adopted group education mode and reported positive effects on improving screening-related outcomes [32,33,35,36,37,38,39]. One study used two types of individualized education and found only the individual home visit showed effective [34]. Therefore, based on these findings we propose that group-based education can be prioritized for consideration in cervical cancer screening health education among females in rural areas. Our findings are inconsistent with a previous systematic review, which showed that individualized education took precedence [22]. However, that review included both urban and rural populations, and most of the included studies were conducted in Western contexts. Our review only focused on rural populations, with most studies conducted in developing countries. Differences in the included participants and study settings might have influenced the results. All healthcare professional-led educational interventions [35,36,37,39,40,41] were effective on improving cervical cancer screening uptake. Regarding the three studies [32,34,38] adopting lay health worker-led education, two [32,34] reported positive changes on the screening uptake rate. However, the resources and ability of training eligible lay health workers in rural areas need to be considered in advanced if adopting lay health workers-involved interventions. Hence, implementing healthcare professional-led approaches could be considered for priority.

## 5. Strengths and Limitations

Regarding the strengths of this review, we studied rural populations from various contexts. Despite diversity among countries, the general characteristics of rural areas are similar [5,6,7]. Gathering studies from various countries/regions to conduct this systematic review could provide evidence on how best to solve this health issue both globally and in individual countries/regions.

This review has some limitations that should be noted. First, we only included studies published in English due to language limitations. As such, only 10 studies were included in this review. The limited number of studies might have contributed to publication bias. It follows that the number of studies pooled into the meta-analysis was small. Because of the limited number of studies, we could not evaluate the publication bias of this review using a funnel plot. Second, we included quasi-experimental studies, which were non-randomized, and this might have caused potential bias. 

## 6. Implications

This review demonstrates that educational interventions for use in rural populations effectively promote the uptake of cervical cancer screening. For the future development of cervical cancer screening-related health education in rural populations, theory-driven, community-involved, group-based, and healthcare professional-led educational approaches are recommended. In terms of the content of cervical cancer screening education, introducing locally available resources and screening services would be beneficial. The influence of sociocultural barriers on behavior in rural areas needs to be assessed to develop culturally tailored educational materials. Meanwhile, to comprehensively evaluate the effectiveness of such interventions, both the short-term and long-term outcomes of educational interventions on cervical cancer screening performance should be examined. 

## 7. Conclusions

Rural health is an essential global public health issue. Due to limited healthcare resources and the high disease burden of cervical cancer in rural areas, rural populations should be considered a vital target for cervical cancer screening. Educational interventions increased the cervical cancer screening uptake rate and knowledge in the rural populations examined in this study. The findings of this review suggest that educational interventions should be developed by theoretical frameworks, and based on the community setting, group sessions, and healthcare professional-led approaches, which could help to promote cervical cancer screening among rural populations. However, the studies included in this review only evaluated the short-term effects of educational interventions. The long-term effects of educational interventions, such as the influence of building regular screening habits among females in rural areas, need to be assessed in future studies. The effects of educational interventions on cervical cancer screening-related self-efficacy, acceptability, and intention still need further investigation.

## Figures and Tables

**Table 1:** Quality appraisal of the RCTs.

	Nuno et al., 2011 [32]	Sossauer et al., 2014 [33]	Thompson et al., 2017 [34]
Q1	U	Y	U
Q2	U	U	U
Q3	Y	Y	Y
Q4	U	U	Y
Q5	U	U	Y
Q6	U	U	Y
Q7	Y	Y	Y
Q8	Y	Y	Y
Q9	Y	Y	Y
Q10	Y	Y	Y
Q11	Y	Y	Y
Q12	Y	Y	Y
Q13	NA	NA	NA
Total score	7	8	10

**Table 2:** Quality appraisal of the quasi-experimental studies.

	Abiodun, 2014 [35]	Choi, 2021 [36]	Eghbal, 2020 [37]	Luque, 2017 [38]	Nagamma, 2020 [39]	Caster, 2017 [40]	Thahirabanuibrahim, 2021 [41]
Q1	Y	Y	Y	Y	Y	Y	Y
Q2	Y	Y	Y	Y	Y	Y	Y
Q3	U	U	Y	Y	Y	NA	NA
Q4	Y	Y	Y	Y	Y	N	N
Q5	Y	Y	Y	Y	Y	Y	Y
Q6	N	Y	Y	Y	Y	Y	Y
Q7	Y	Y	Y	Y	Y	Y	Y
Q8	Y	Y	Y	Y	Y	Y	Y
Q9	Y	Y	Y	Y	Y	Y	Y
Total score	7	8	9	9	9	7	7

**Table 3:** Data extraction of the included studies.

Study	Study Design	Population				Screening	Intervention	Comparison	Follow-Up	
		Target People	Financial Situation	Educational Background	Sample Size (IG/CG)				Duration	Withdraw (IG/CG)
Nuño et al., 2011, USA [32]	RCT	50–66 years and even older	Average monthly income: IG: $895 CG: $933	77.4% in elementary school or lower	381 (190/191)	Pap smear	Promotora- administered group education	Usual care	2 years	10 (7/3)
Sossauer et al., 2014, Cameroon [33]	RCT	25–65 years	Not report	High school graduate took the largest account (56.3%)	302 (152/150)	HPV self-sampling	Educational intervention	Usual care	Immediately after the intervention	1 (0/1)
Thompson et al., 2017, USA [34]	RCT (Three-armed)	21–64 years; have not had a Pap test within the past 3 years	24.9% had health insurance now, 18.3% never had	93.2% in high school or lower	443 (296/147)	Pap smear	A: culturally appropriate video B: culturally appropriate in home Promotora-led educational intervention	Usual care	7 months	40 (28/12)
Abiodun et al., 2014, Nigeria [35]	QE (with a control group)	25–64 years	Average monthly income: 76.9% lower than 15,000 Naira (lower than $40)	86.9% in secondary school or lower	700 (350/350)	VIA	Multiple media health education based on a movie	Breast cancer education	13 weeks	86 (25/61)
Choi and Kim, 2021, USA [36]	QE (with a control group)	21–65 years, have not had a Pap test within the past 3 years	Most (97.8%) had health insurance	College/university graduate took the largest account (73.9%)	46 (20/26)	Pap smear	Cervical cancer prevention education program	Not report	8 weeks	0
Eghbal et al., 2020, Iran [37]	QE (with a control group)	20–65 years and married at least once	Not report	67.5% in elementary or lower	160 (80/80)	Pap smear	Educational program	Usual care	2 months	0
Luque et al., 2017, USA [38]	QE (with a control group)	22–62 years and had not received a Pap test in 2 years or more	Median weekly income: $250–$500	Not report	90 (38/52)	Pap test	Salud es Vida	Nutrition class	6 months	0
Nagamma et al., 2020, India [39]	QE (with a control group)	18–55 years	Not report	Secondary graduate took the largest account (43.4%)	166 (82/84)	Pap smear	Audio-visual media intervention	Pamphlet	Immediately after the intervention	0
Caster et al., 2017, Malawi [40]	QE (single group pre-post test)	18–77 years	Monthly income: most (74%) less than $42	Standard 4–8 took the largest account (46%)	243 (117 in pre-and post-test group)	Not report	Tablet-based education program	Immediately after the intervention	——	——
Thahirabanuibrahim and Loga raj, 2021, India [41]	QE (single group pre-post test)	30–60 years	Lower class took the largest account (32.43%)	Primary graduate took the largest account (32.4%)	370	Pap smear	Health education model	Not report	——	——

IG: Intervention group; CG: Control group; QE: Quasi-experimental study.

**Table 4:** Intervention components.

Study	Intervention	Intervention Components					
		Theoretical Framework	Delivery Mode	Dosage		Intervener	Settings
				Duration	Frequency		
Nuño et al., 2011, USA [32]	Promotora-administered group education	Social Cognitive Theory	Face-to-face group educational sessions: 3–12 women in one group	2 h a session	Participants needed to attend at least one class	Promotora	Community (participants’ home)
Sossauer et al., 2014, Cameroon [33]	Educational intervention	Not report	Face-to-face group education	Discussion: 5 min, Video: 6 min	Not report	Healthcare professional (research team)	Healthcare center
Thompson et al., 2017, USA [34]	A: culturally appropriate video B: culturally appropriate in home Promotora-led educational intervention	Social Cognitive Theory	A: Self-directed learning: watching video B: Individual home visit	A: video: 13 min B: Not report	A: Not report B: once	A: self-direct learning B: Promotora	Community (participants’ home)
Abiodun et al., 2014, Nigeria [35]	Multiple media health education based on a movie	Not report	Face-to-face group educational sessions, 50 women in one group	More than 4 h a day	7 days	Healthcare professional (research team)	Not report
Choi and Kim, 2021 USA [36]	Cervical cancer prevention education program	Theory of Planned Behavior	Face-to-face group educational sessions	1 h a session	Once a week for 4 weeks	Healthcare professional (research team)	Community (church)
Eghbal et al., 2020, Iran [37]	Educational program	Health Belief Model	Face-to-face group educational sessions	50–60 min each session	Once a week for 3 weeks	Healthcare professional (research team)	Healthcare center
Luque et al., 2017, USA [38]	Salud es Vida	Social Cognitive Theory and Popular Education	Face-to-face group educational sessions, an average of 7 women in one group	3 h each class	A total of 17 classes held with small groups	Promotora	Community (public places in the community and individual homes)
Nagamma et al., 2020, India [39]	Audio-visual media intervention	Not reported	A: Face-to-face group educational session	A: 30 min	A: seven sessions	Healthcare professional (research team)	Community
Caster et al., 2017, Malawi [40]	Tablet-based education program	Not report	Tablet	30 min	Once	Healthcare professional (research team)	Not reported
Thahirabanuibrahim and Logaraj, 2021, India [41]	Health education model	Theory of Planned Behavior	Video presentation	Not report	Once	Healthcare professional (research team)	Community

**Table 5:** Educational content.

Author, Year	Basic Knowledge	Psychological Issues	Barriers to Screening and Overcoming Strategies	Locally Available Resources
Nuño et al., 2011 [32]	ü	ü		ü
Sossauer et al., 2014 [33]	ü	ü		
Thompson et al., 2017 [34]	ü		ü	ü
Abiodun, et al., 2014 [35]	ü			
Choi and Kim, 2021 [36]	ü			ü
Eghbal et al., 2020 [37]	ü	ü	ü	ü
Luque et al., 2017 [38]	ü		ü	ü
Nagamma et al., 2020 [39]	ü			
Caster et al., 2017 [40]	ü			ü
Thahirabanuibrahim and Logaraj, 2021 [41]	ü			ü

**Table 6:** Educational materials.

Author, Year	Audio-Visual Materials	Reading Materials
	Video/Audio	Leaflet/Brochure/Pamphlet/Booklet
Nuño et al., 2011 [32]	Not report	
Sossauer et al., 2014 [33]	ü	
Thompson et al., 2017 [34]	ü	ü
Abiodun, et al., 2014 [35]	ü	ü
Choi and Kim, 2021 [36]	Not report	
Eghbal et al., 2020 [37]	ü	ü
Luque et al., 2017 [38]	ü	ü
Nagamma et al., 2020 [39]	ü	ü
Caster et al., 2017 [40]	ü	
Thahirabanuibrahim and Logaraj, 2021 [41]	ü	

**Table 7:** Studies’ results and conclusions.

Author, Year, Study Site	Outcomes	Conclusion
Nuño et al., 2011, USA [32]	Uptake: post-intervention 89% of rural females in IG got screening while in CG it was 75% (*p* < 0.01)	A Promotora-based educational intervention effective on increasing cervical cancer screening uptake
Sossauer et al., 2014, Cameroon [33]	Knowledge: post-intervention 81.6% of rural females in IG got good knowledge while in CG it was 10.1% (*p* < 0.01) Acceptability: no significant difference in post-intervention level between the IG and CG Willingness: no significant difference in post-intervention level between the IG and CG	Educational intervention effective on increasing knowledge about HPV and cervical cancer, but not on Self-HPV acceptability
Thompson et al., 2017, USA [34]	Uptake: post-intervention 53.4% of rural females in IG B got screening, while in CG it was 34.0% (*p* < 0.01); No significant difference between IG A and CG Knowledge: post-intervention correct response rate of IG A was 40.7%; IG B was 36.7%; CG was 26.7% (*p* < 0.05) Self-efficacy: no significant difference in post-intervention self-efficacy level between the IG and CG	Culturally appropriate in-home Promotora-led educational intervention successful in increasing cervical cancer screening
Abiodun et al., 2014, Nigeria [35]	Uptake: post-intervention 8.3% of rural females in IG got screening while in CG it was 3.8% (*p* < 0.05) Knowledge: post-intervention score of IG was (25.69 ± 6.20), and CG was (2.22 ± 6.04) (*p* < 0.01) Awareness: post-intervention 100% rural females in IG aware while in CG it was 10.7% (*p* < 0.01) Perception: post-intervention score of IG was (4.43 ± 0.92), and CG was (1.17 ± 0.88) (*p* < 0.01) Willingness: no significant difference in post-intervention willingness between the IG and CG	Multiple media health education based on a movie effective in creating awareness and improving the knowledge, perception, and uptake of cervical cancer screening
Choi and Kim, 2021, USA [36]	Uptake: post-intervention 35.5% of rural females in IG got screening while in CG it was 7.7% (*p* < 0.05)	Cervical cancer prevention education program effective on increase Pap screening rate
Eghbal et al., 2020, Iran [37]	Uptake: IG increased from 18.75% to 78.75% while CG increased from 16.25% to 22.5% (*p* < 0.01) Knowledge: post-intervention score of IG was (25.2 ± 2.1), and CG was (19.7 ± 1.6) (*p* < 0.01) Self-efficacy: post-intervention score of IG was (24.7 ± 1.0), and CG was (19.1 ± 3.0) (*p* < 0.01)	Educational program effective on increasing cervical cancer screening behavior among rural women
Luque et al., 2017, USA [38]	Uptake: no significant difference between IG and CG Knowledge: post-intervention score of IG was (11.5 ± 2.1), and CG was (10.7 ± 1.7) (*p* < 0.05) Self-efficacy: no significant difference in post-intervention self-efficacy between the IG and CG	Group educational intervention associated with increased cervical cancer knowledge, but not uptake of Pap test
Nagamma et al., 2020, India [39]	Knowledge: post-intervention knowledge about Pap smear increased in both two groups (*p* < 0.01)	Face-to-face interactive sessions positive on increasing cervical cancer-related knowledge
Caster et al., 2017, Malawi [40]	Knowledge: post-test correct respond about screening increased (*p* < 0.01) Desire: post-test 93% participants showed a desire for cervical cancer screening	Tablet-based educational program effective, feasible and acceptable to disseminate cervical cancer information
Thahirabanuibrahim and Logaraj, 2021, India [41]	Knowledge: pre-test score 1.34, post-test score 2.34 (*p* < 0.05) Attitude: pre-test score 1.11, post-test score 1.96 (*p* < 0.01) Uptake: post-test: 30.1% participants finished the screening	Health education model proved to be efficacious on cervical cancer prevention

## Data Availability

The data presented in this study are available in this article and its Supplementary Materials.

## Supplementary Materials

The following supporting information can be downloaded at: https://www.mdpi.com/article/10.3390/ijerph19116874/s1.
