# Supplementary material for: Educational Interventions to Promote Cervical Cancer Screening among Rural Populations: A Systematic Review
Source: Int J Environ Res Public Health. 2022 Jun 4;19(11):6874. doi: 10.3390/ijerph19116874 (PMC9180749; doi:10.3390/ijerph19116874)
Supplement: Supplementary file 1 [file ijerph-19-06874-s001.zip › ijerph-1654346-supplementary.pdf]

MEDLINE	Search statement	Result
1	((uterine or cerv*) and (cancer or neoplasm*)).mp.	172419
2	early detection of cancer/	31833
3	(screen* or test*).mp.	4497774
4	2 or 3	4504351
5	(pap* and (test or smear)).mp.	71806
6	(thinprep cytolog* test or TCT).mp.	1350
7	(HPV and (test* or samp*)).mp.	17475
8	5 or 6 or 7	83784
9	4 or 8	4510563
10	1 and 9	42553
11	educat*.mp.	1025632
12	(uptak* or atten* or particip* or adheren*).mp.	2571548
13	knowledge.mp.	730002
14	(attitude or awareness or perception or accep* or intention or will*).mp.	2270943
15	12 or 13 or 14	4805385
16	10 and 11 and 15	2586
17	(rural or countryside* or village).mp.	175314
18	16 and 17	274

EMBASE	Search statement	Result
1	((uterine or cerv*) and (cancer or neoplasm*)).mp.	199137
2	early detection of cancer/	8121
3	(screen* or test*).mp.	6613655
4	2 or 3	6613655
5	(pap* and (test or smear)).mp.	196509
6	(thinprep cytolog* test or TCT).mp.	4338
7	(HPV and (test* or samp*)).mp.	29750
8	5 or 6 or 7	215890
9	4 or 8	6628725
10	1 and 9	65297
11	educat*.mp.	1450714
12	(uptak* or atten* or particip* or adheren*).mp.	3869234
13	knowledge.mp.	1034754
14	(attitude or awareness or perception or accep* or intention or will*).mp.	3710416
15	12 or 13 or 14	7410430
16	10 and 11 and 15	4196
17	(rural or countryside* or village).mp.	231155
18	16 and 17	491

PubMed	Search statement	Result
1	((uterine or cerv*) and (cancer or neoplasm*))	212599
2	early detection of cancer[MeSH Terms]	31819
3	screen*[Title/Abstract] OR test*[Title/Abstract]	4192380
4	2 or 3	4200003
5	(pap or papanicolaou) and (test or smear)	16024
6	thinprep cytolog* test or TCT	3034
7	HPV and (test* or samp*)	19997
8	5 or 6 or 7	35313
9	4 or 8	4212003
10	1 and 9	46699
11	educat*[Title/Abstract]	697887
12	uptak* or atten* or particip* or adheren*	3066432
13	knowledge	901909
14	attitude or awareness or perception or accep* or intention or will*	3514630
15	12 or 13 or 14	6298216
16	10 and 11 and 15	2656
17	rural or countryside* or village	249159
18	16 and 17	315

Web of Science	Search statement	Result
1	TS=((cerv* OR uterine) AND (cancer OR neoplasm*))	119203
2	TS=(early detection of cancer)	50317
3	TS=(screen* OR test*)	7247097
4	2 or 3	7276398
5	TS=((pap OR papanicolaou) AND (test or smear))	16006
6	TS=(thinprep cytolog* test or TCT)	3673
7	TS=(HPV and (test* or samp*))	19664
8	5 or 6 or 7	35273
9	4 or 8	7286563
10	1 and 9	38346
11	TS=educat*	1612320
12	ALL=(uptak* or atten* or particip* or adheren*)	4772596
13	ALL=(knowledge)	2050966
14	ALL=(attitude or awareness or perception or accep* or intention or will*)	6617638
15	12 or 13 or 14	11688768
16	10 and 11 and 15	2600
17	ALL=(rural or countryside* or village)	611776
18	16 and 17	342

CINAHL	Search statement	Result
1	AB (uterine or cerv*) AND AB (cancer or neoplasm*)	15771
2	TX early detection of cancer	23200
3	AB screen* OR AB test*	733948
4	2 OR 3	747071
5	TX (pap or papanicolaou) AND TX (test or smear)	14401
6	TX thinprep cytolog* test OR TX TCT	1426
7	TX HPV AND TX (test* or samp*)	11872
8	5 OR 6 OR 7	24340
9	4 OR 8	761740
10	1 AND 9	8386
11	AB educat*	332552
12	TX uptak* or atten* or particip* or adheren*	1620701
13	TX knowledge	674395
14	TX attitude or awareness or perception or accep* or intention or will*	2639893
15	12 OR 13 OR 14	3340060
16	10 AND 11 AND 15	1323
17	TX rural or countryside* or village	204241
18	16 AND 17	271

Cochrane	Search statement	Result
1	(cerv* OR uterine) AND (cancer OR neoplasm*)	9347
2	MeSH descriptor: [Early Detection of Cancer] explode all trees	1384
3	screen* OR test*	470453
4	2 OR 3	470540
5	(pap OR papanicolaou) AND (test OR smear)	1767
6	thinprep cytolog* test OR TCT	1187
7	HPV AND (test* OR samp*)	1741
8	5 OR 6 OR 7	4344
9	4 OR 8	471814
10	1 AND 9	3650
11	educat*	106893
12	uptak* or atten* or particip* or adheren*	427090
13	knowledge	48893
14	attitude or awareness or perception or accep* or intention or will*	411790
15	12 OR 13 OR 14	674972
16	10 AND 11 AND 15	643
17	rural or countryside* or village	12577
18	16 AND 17	94
19	Searched in Cochrane Central Register of Controlled Trials	48

Global Health	Search statement	Result
1	((uterine or cerv*) and (cancer or neoplasm*)).mp.	23853
2	early detection of cancer.mp.	148
3	(screen* or test*).mp.	835310
4	2 or 3	835353
5	(pap* and (test or smear)).mp.	23226
6	(thinprep cytolog* test or TCT).mp.	386
7	(HPV and (test* or samp*)).mp.	10595
8	5 or 6 or 7	30091
9	4 or 8	838517
10	1 and 9	13407
11	educat*.mp.	192543
12	(uptak* or atten* or particip* or adheren*).mp.	531920
13	knowledge.mp.	159067
14	(attitude or awareness or perception or accep* or intention or will*).mp.	352807
15	12 or 13 or 14	887434
16	10 and 11 and 15	1298
17	(rural or countryside* or village).mp.	117133
18	16 and 17	169

Clinical trials	Search statement	Result
1	Cervical cancer screening AND education AND rural	61
